# Milk protein-based active edible packaging for food applications: An eco-friendly approach

**DOI:** 10.3389/fnut.2022.942524

**Published:** 2022-07-26

**Authors:** Vandana Chaudhary, Priyanka Kajla, Parveen Kumari, Sneh Punia Bangar, Alexandru Rusu, Monica Trif, Jose M. Lorenzo

**Affiliations:** ^1^Department of Dairy Technology, College of Dairy Science and Technology, Lala Lajpat Rai University of Veterinary and Animal Sciences, Hisar, Haryana, India; ^2^Department of Food Technology, Guru Jambheshwar University of Science and Technology, Hisar, Haryana, India; ^3^Department of Food, Nutrition and Packaging Sciences, Clemson University, Clemson, SC, United States; ^4^Department of Food Science, Life Science Institute, University of Agricultural Sciences and Veterinary Medicine Cluj-Napoca, Cluj-Napoca, Romania; ^5^Food Research Department, Centre for Innovative Process Engineering (CENTIV) GmbH, Stuhr, Germany; ^6^Centro Tecnológico de la Carne de Galicia, Ourense, Spain; ^7^Área de Tecnología de los Alimentos, Facultad de Ciencias de Ourense, Universidade de Vigo, Ourense, Spain

**Keywords:** active packaging, edible packaging, milk proteins, encapsulation, functional properties

## Abstract

Whey and casein proteins, in particular, have shown considerable promise in replacing fossil-based plastics in a variety of food applications, such as for O_2_ susceptible foods, thereby, rendering milk proteins certainly one of the most quality-assured biopolymers in the packaging discipline. Properties like excellent gas barrier properties, proficiency to develop self-supporting films, adequate availability, and superb biodegradability have aroused great attention toward whey and other milk proteins in recent years. High thermal stability, non-toxicity, the ability to form strong inter cross-links, and micelle formation, all these attributes make it a suitable material for outstanding biodegradability. The unique structural and functional properties of milk proteins make them a suitable candidate for tailoring novel active package techniques for satisfying the needs of the food and nutraceutical industries. Milk proteins, especially whey proteins, serve as excellent carriers of various ingredients which are incorporated in films/coatings to strengthen barrier properties and enhance functional properties viz. antioxidant and antimicrobial. In this review, the latest techniques pertaining to the conceptualization of active package models/ systems using milk proteins have been discussed. Physical and other functional properties of milk protein-based active packaging systems are also reviewed. This review provides an overview of recent applications of milk protein-sourced active edible packages in the food packaging business.

## Introduction

There is a rising fascination with innovative packaging systems developed from edible biopolymers such as proteins, carbohydrates, and lipids that are biodegradable, environmentally safe, and a viable alternative to petroleum-based materials commonly used in the food industry. Furthermore, packaging materials must have trustworthy characteristics such as non-toxicity, microbiological stability, excellent physical properties in terms of mechanical, barrier properties, good organoleptic characteristics, minimal manufacturing expenses, and be completely compatible with the packaged product (1). The surplus availability of whey and other by-products of the milk processing segment has aroused substantial curiosity among the packaging industries to utilize these as major ingredients of edible films/coatings. Milk proteins, especially whey and casein, are reported to have all the essential ingredients required for a perfect biodegradable and biocompatible film/coating formulation (2). Milk protein is made up of 80 % casein and 20% whey protein, which has been segregated into various components. Whey proteins are the most economically and technically intriguing ingredient of whey, accounting for around 15–20% of total milk proteins. Henceforth, milk proteins either whey or casein alone or in combination can be used to develop edible food coatings having different physical and functional properties (3). For a stable and quality edible film formation of a three-dimensional network is required which involves biopolymer-based (protein-protein) interactions. Along with nutritional benefits, casein and whey proteins have versatile physicomechanical properties *viz*. solubility, emulsification, and biodegradability that make them ideal for use in edible films (4). Packaging systems sourced from dairy protein serve as an excellent shield against physical and microbial contamination of the food and thereby play important role in shelf-life extension and quality maintenance of varied food products. The deliquescent nature of milk proteins and the cross-linked interactions betwixt protein chains help in the formation of densely packed three-dimensional network structures (5). The three-dimensional complexed structure of milk proteins makes excellent coatings/films having greater stability longer, durability, and excellent barrier properties in comparison to polysaccharide-based coatings (2).

**Figure 1:**
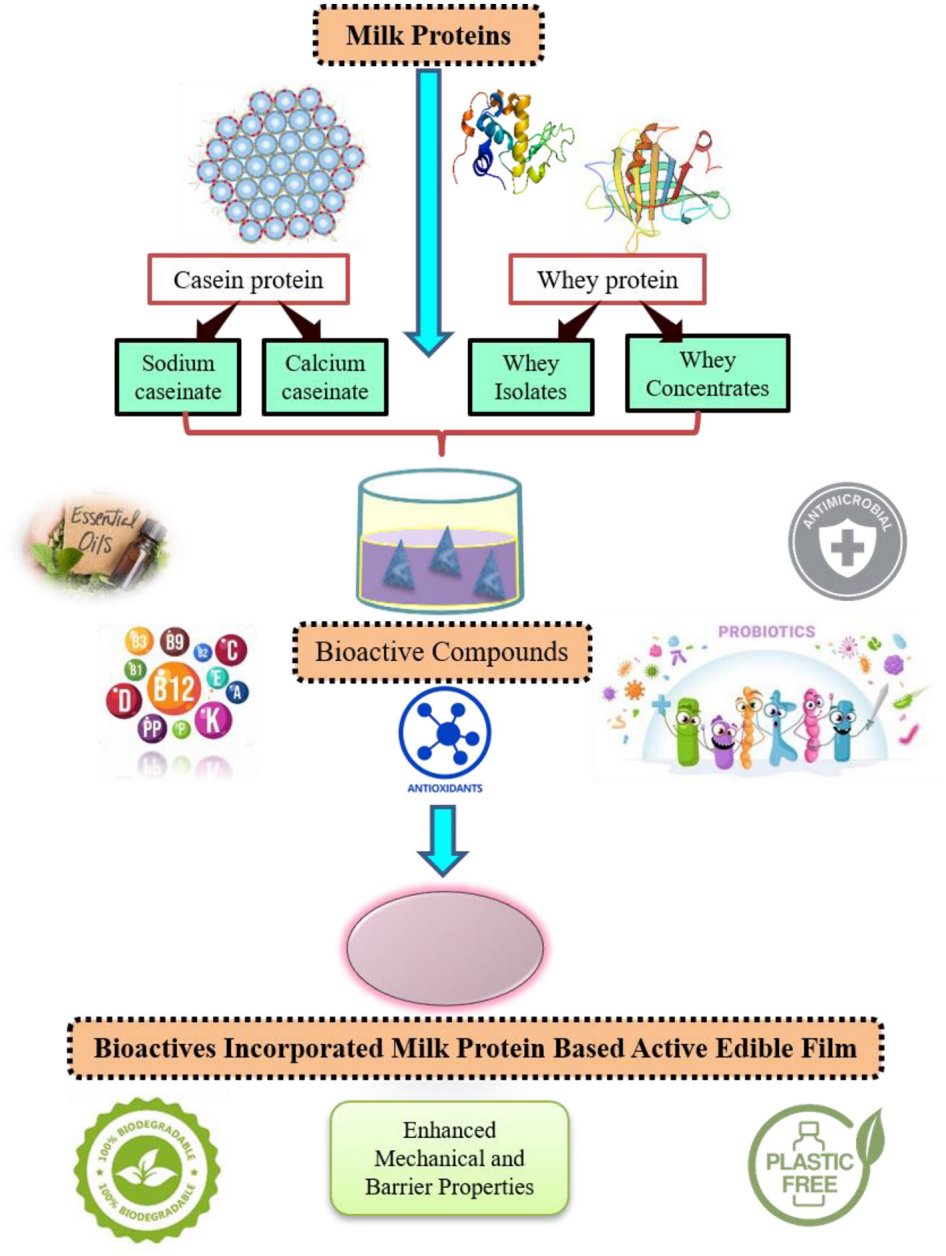
Techno-functional characteristics of milk protein-based active edible packaging system.

Non-toxicity, biodegradability, safe to use, biocompatibility with different food products as well as external environment, along with phenomenal functional attributes *viz*. stability, water-binding capacity, emulsification property, gelation and foaming ability make milk proteins exemplary components for the development of different innovative types of novel food packaging systems (6). These different structural and functional peculiarities of dairy proteins expedite the development/customization of edible film/coating alone or in consolidation with instinctive natural/bioactive materials in the form of hydrogels, micro or nanocapsules, nanocomposites with a myriad of assuring quality characteristics for food, pharmaceuticals as well as biotechnological applications which have application at the macro, micro, and nano levels as hydrogels and micro- or nanocapsules, and floating beads (7–9). Milk proteins are compatible with food systems containing bioactive substances due to their unique protein structure. As a result, biologically active and nutraceutical chemicals can be coupled with milk proteins to create active packaging systems that are compatible, biodegradable, eco-friendly as well as manage bio-accessibility and active ingredient delivery/release at the target site (10). Dairy proteins also have additional functionalities in comparison to normal packaging materials as these proteins exhibit antimicrobial and immunomodulatory functions (11) (Figure 1).

Mechanical and barrier properties of edible films/coatings are expected to maintain package durability and quality as well as monitoring of mass transfer characteristics to restrict the diffusion/permeate of different external and internal constituents in either direction which aids in preserving sensory and along with other quality attributes of packed products (2). Whey and casein protein-based films outplay polysaccharides and other protein-source-based films in terms of physical as well as functional properties. Whey protein-based edible films/coatings have found applications in the food arena as several studies investigated the usage of these films in different categories of food products *viz*. breakfast cereals, fruits and vegetables, animal origin-based foods as well as processed milk products to ameliorate different properties like improved water and gas barriers property, preserving of flavor and aromatic compounds. Based on literature studies, milk proteins could be ideal carriers for different nutraceuticals/bioactive compounds, vitamins, and minerals using micro/nanoencapsulation techniques owing to superb compatibility, ease of controlled release at the target site, high solubility, and most importantly, ease of degradation (12). Furthermore, incorporating active ingredients *viz*. antimicrobials, antioxidants, probiotics/prebiotics, and polyphenols into milk protein films/coatings is a new trend in the industry targeting to provide health benefits to the consumers. So, this review compiles the latest trends regarding the techniques used to formulate active packaging using milk proteins, their barrier, mechanical, and other important functional properties. Authors have compiled comprehensive food applications and future prospects of milk protein-based coatings/films.

## Milk proteins (whey and casein) as a film-forming material

Milk is a nutrient-dense food with a conglomeration of various macro-and micronutrients vital for human health. Milk proteins possess a wide array of structural and functional characteristics that can help produce macro, micro, and nanostructures with a variety of intriguing properties for culinary and biotechnological relevance. Their nutritional makeup, environment-friendly nature, non-toxic, biocompatibility, excellent thermostability, gelation, emulsification, water-binding capacity, and foaming, to name a few characteristics, all encourage their application in the designing of novel packaging systems for food products (6). Total bovine milk proteins encompass ~80% casein and 20% whey proteins. Four major casein constituents are αs1-, αs2-, β-, κ-casein, and a minor component is γ-casein. The typical concentration of these casein constituents is 38, 10, 36, 13, and 3%, respectively (2). In general, casein exists in the form of aggregated structures known as micelles which are further stabilized by calcium phosphate coupling. Moreover, caseins are low in cysteine resulting in less disulfide bonding leading to an exposed, arbitrary structure (13). A biopolymer, sodium caseinate annexed by acid precipitation of casein is used as an alternative to conventional petroleum-based packaging (14). It possesses superior film-forming ability owing to its random coil structure and its capability to form a considerable number of intermolecular bonds *via* hydrophobic, electrostatic interactions, and hydrogen bonds (15, 16). As observed by Jahromi and his team, the dissolution rate in water and elongation at the break of edible films increased from 27.16 to 63.70% and 2.36 to 16.53%, respectively, when the calcium caseinate and high methoxy pectin ratio was changed from 100:0 to 25:75. Also, the glass transition temperature of high methoxy pectin-integrated caseinate films was lower (17). No doubt, sodium caseinate exhibits good film-forming characteristics but these films have lower mechanical strength because of hydrophilic residues. To overcome this limitation, a crosslinker, Genipin, was added to the film-forming solution. The films so produced had much higher Young's modulus and tensile strength and significantly lower break elongation (18). Caseinates have high heat stability, which restricts probable changes during thermal processing, which might be beneficial in the development of innovative packaging materials. For instance, the thermal properties of collagen fiber-based films enhanced significantly after crosslinking with 50% casein (19). Crosslinking of caseinates with divalent cation calcium had improved moisture barrier and mechanical properties. This may be attributed to the open structure of the casein coil and its tendency to establish intermolecular linkages by the formation of hydrogen and hydrophobic bonds leading to a more stable structure (20).

Whey is obtained as a by-product of the cheese industry or during the acid-assisted coagulation of casein. Whey is mainly constituted of lactose, succeeded by proteins, lactic acid, and lipids. Whey proteins (15–20%) of the total milk proteins consists of β-lactoglobulin (β-Lg) (~57%), α-lactalbumin (α-La) (~19%), several immunoglobulins (~13%), bovine serum albumin (~7%), and the polypeptides proteose-peptone (~4%) and are globular. Being the most abundant whey protein β-Lg drives the aggregation and gelation characteristics of whey-based formulations (21). Because of its compact globular form and small molecular size, whey protein cannot be regarded as a good adhesive polymer choice. Under specific conditions, however, the globular structure can be reorganized into somewhat straight structures and then into unreversible agglomerates *via* thiol-disulfide exchange (5). The abundance of whey protein and its potential to make foams, gels, emulsions, and biogenic material piques the interest of the packaging industry, allowing its usage as films/coatings on the external surface of food, to shield goods from chemical or microbial degradation, hence extending shelf life and maintaining excellent product standards (22). Whey protein films are garnering increased attention for their biodegradable and edible nature; mechanical and barrier qualities as they surpass polysaccharides and other protein-based films derived from other sources and also as a carrier for bioactive components in active films (23, 24). The embodiment of silver nanoparticles in whey protein concentrates edible films ameliorated the moisture impermeability by 67% as well as the tensile strength by 84% of the films. Furthermore, the films appreciably inhibited the growth of food pathogens like *Escherichia coli* O157:H7, *Staphylococcus aureus, Listeria monocytogenes, Salmonella enteritidis*, and *Aspergillus sydowii* displaying 13 to 19.7 mm zones of inhibition (25). The antibacterial, antioxidant, and physicochemical attributes of whey protein-based edible films including various soy sauces were studied by García et al. (26). A convincing difference in mechanical characteristics was not observed but there was an appreciable enhancement in film elongation. The application of these films was able to restrict the growth of food-spoiling bacteria *L. monocytongenes, E. coli*, and *S. Typhimurium*. Characteristics of the edible films are dependent on the type of milk proteins. For instance, hydrodynamic (moisture content, water solubility, swelling ratio, water vapor permeability), mechanical (thickness, tensile strength, elongation at break), color, and antioxidant (DPPH) properties of edible films made from casein and whey protein isolates were examined in this study (two types, WPI1 and WPI2). Casein-based films revealed elevated values of moisture content (40.21%), thickness (0.193 mm), elongation at break (49.67%), and antioxidant capacity (32.64 % DPPH inhibition), but had curtailed water vapor permeability value of 15.28 g/m^2^day (27).

## Technologies to prepare milk protein-based active edible packaging

**Figure 2:**
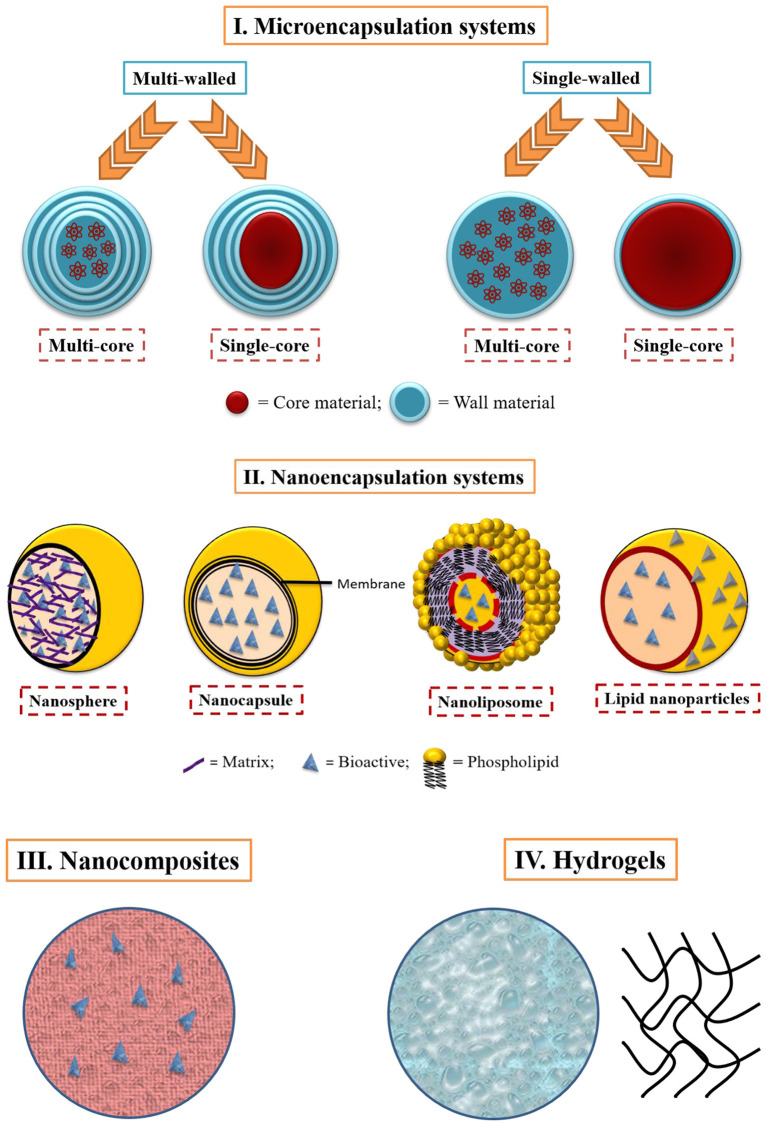
Milk protein based micro-, nanoparticles, nanocomposites, and hydrogels (38).

Active packaging is a novel method of preserving or lengthening the storage life of food products and retaining their integrity, freshness, safety, and purity. Active packaging may be referred to as “the packaging systems that intentionally integrate compounds that would liberate or devour compounds into or from the packed food or the environment encompassing the food,” as defined by the European regulation (EC) No. 450/2009 (28). The traditional method of active packaging production entails adding bioactive compounds to the film-forming solution succeeded by casting the film (29). Alternatively, the active substance can also be encapsulated with colloidal particles before being mixed with the film-forming solution (30). The transition of active substances from the micro- to nanoscale opens up new possibilities for the food processing sector. Milk proteins serve as impeccable vehicles for micro- or nanoencapsulated bioactive compounds like nutraceuticals, antimicrobial, antioxidant compounds, etc. by virtue of their sustained release, biocompatibility and ease of dispersibility of encapsulated bioactives (12, 31). Milk proteins have versatile properties and can be applied to create microparticles (32, 33), nanoparticles (34, 35), nanocomposites (36), nanocapsules, hydrogels (37), etc. either independently or in a combination of other biopolymers (Figure 2).

**Figure 3:**
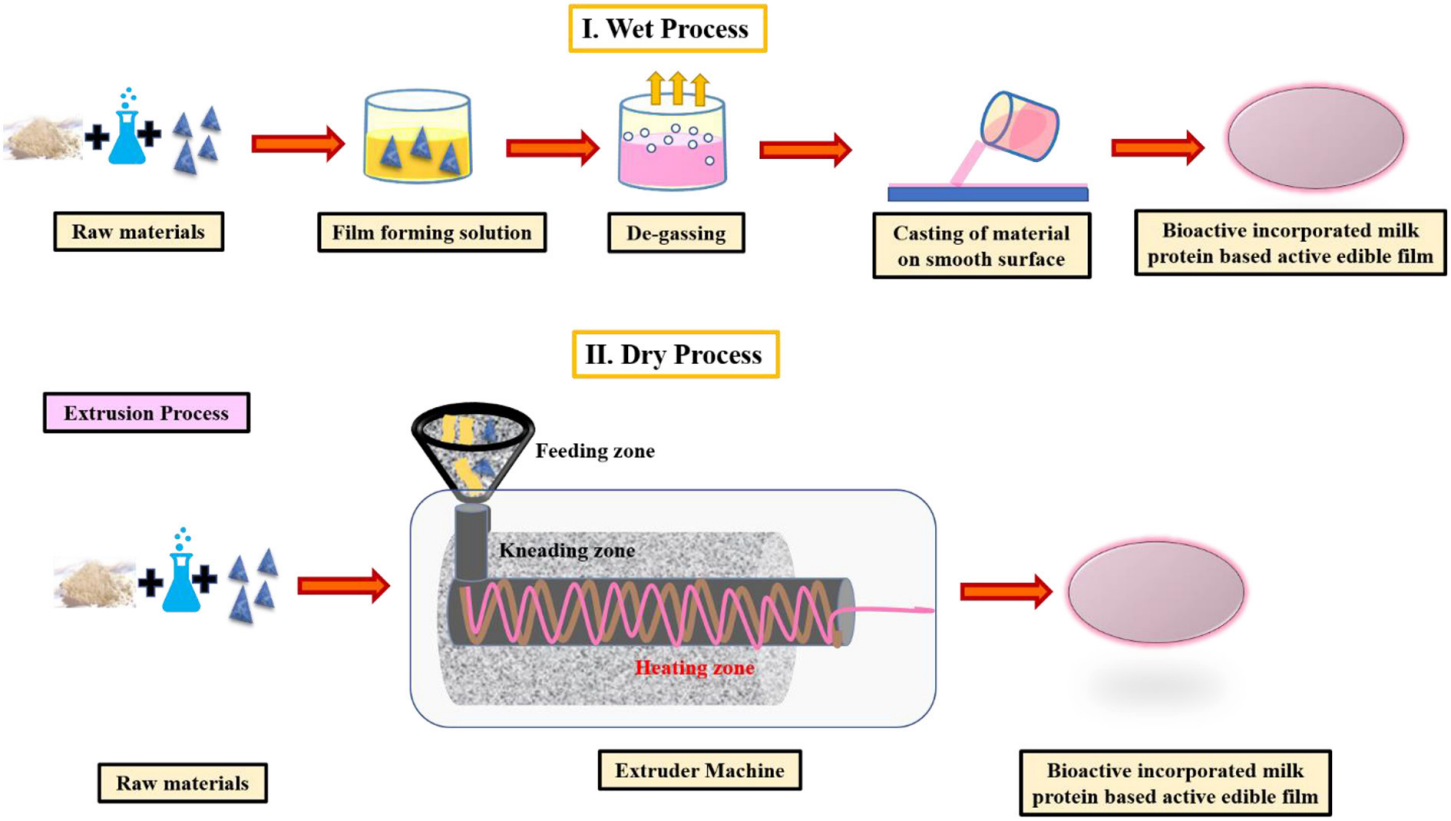
Wet and dry process (e.g., extrusion) for production of milk protein based active edible films (40).

The conventional methods for the production of edible films have been split into two categories: wet and dry processes. In the wet process, solvents are required for the solubility and spreading of the polymer on a smooth and flat plane, further followed by drying under controlled conditions to remove the solvent. It is an energy-intensive method that is more suitable for laboratories but finds its limited usage on a commercial scale. Injection, blow-molding, extrusion, and heat-pressing are among the most regularly used dry methods for producing edible films (39) (Figure 3). Generally, the method employed for the formation of milk protein films employs the denaturation of milk proteins at a temperature range of 75–100°C leading to the production of intermolecular disulfide bonds which might be held accountable for the structure of the film. Dissemination of charged, polar, and non-polar amino acids alongside the chain of proteins with the resultant interactive forces aid in the configuration of a coherent matrix of proteins as a result of varied functional groups of amino acids, providing multitudinal locus for interaction, thereby leading to enhanced film characteristics (41, 42). Other components can be appended pre- or post-heating, depending on their congeniality with each other. Heat-tolerant compounds like starch and prebiotics can be added at the beginning of the edible active film whereas heat-labile components like probiotics, antimicrobial compounds, antioxidants, and some vitamins are added after the heating is concluded (43).

## Impact of milk proteins on the properties of active edible films

Techno-functional properties of milk protein based active edible films are briefly discussed in this section. Recent research findings pertaining to mechanical properties, moisture and gas restriction properties, optical properties as well as antioxidant and antimicrobial properties are presented in Table 1.

**Table 1:** Mechanical, barrier, and functional properties of milk protein-based active packaging materials.

**Milk protein**	**Active material**	**Beneficial effects on properties**	**References**
Whey protein isolate	Pomegranate seed oil (0.3%w/v)	↑Elongation at break, ↓ transparency, ↓WVP, and poor solubility	(44)
Whey protein concentrate	Essential oils (Rosemary and cinnamon)	↓Solubility, ↑WVTR	(45)
Whey protein isolate	Tarragon essential oil	↑Transmittance values, ↑ tensile strength, ↑Elongation at break, ↑ antimicrobial activity	(46)
Whey proteins	Oregano oil	↑ WVP, ↑ flexibility, ↑ moisture barrier properties and ↑ antimicrobial activity	(24)
Casein	Chitosan	↑WVP, ↑film solubility, ↑ antimicrobial activity against *E. coli, Staphylococcus aureus*	(47)
Whey protein concentrate	Pectin/alginate	↓Viscosity, ↓ water affinity, ↓ mechanical strength, ↓ barrier values, ↑opacity, ↑thermal stability	(48)
Whey protein isolate	Pectin	↑Opacity, ↑WVP, and ↑solubility, ↓flexibility, ↓tensile strength	(49)
Whey protein concentrate	Nanocrystalline cellulose and transglutaminase	↑Tensile strength, ↑elongation properties	(50)
Calcium caseinate + Whey proteins	Carboxymethylcellulose	↑ Oxygen barrier, ↑ antioxidant property	(51)
Casein	Tannins	↑ Antioxidant activity, ↑ antimicrobial activity, ↓ water solubility, ↓ water vapor permeability, ↓ stretchability, ↑ thickness	(52)

### Mechanical properties

Packaging materials must have adequate mechanical properties and flexibility to maintain package coherence and have enough endurance to absorb external forces during the production, handling, and storage of packed products. The formulation and processing techniques used in film development determine the mechanical properties of whey protein-based films and coatings. The mechanical properties of whey milk protein-based films are substantially determined by two factors. The first is the formation of a three-dimensional protein network while the film-forming process. Materials with a dense cross-linked network result in the formulation of a durable and strong coating/film having increased modulus and strength. The second factor is the presence of plasticizers. The use of plasticizers reduces the number of intermolecular interactions between protein chains. The plasticizer type, as well as the ratio, has an impact on mechanical performance (27, 53).

Plasticized glycerol with edible films was reported to have increased elongations but decreased tensile strength, followed by other plasticizers viz. sucrose and polyethylene glycol. On the contrary, whey protein isolate films plasticized with glycerol had improved mechanical properties in comparison to the films containing sorbitol and xylitol as plasticizers (23). Based on these findings, it can be stated that glycerol as a plasticizer acts as a decisive factor in defining the physical properties of protein-sourced films as it lowers the intermolecular tensions among different polymers by increasing elongation at break and decrement in tensile strength. Furthermore, cavities and holes may reduce the extensibility and mechanical strength of the film or coating. As a result, protein-based packaging ought to have efficient mechanical properties in order to safeguard food while handling and storage (27).

The incorporation of unmodified Na+-montmorillonite into whey protein-based films resulted in increased tensile strength and decrement in the elongation at break (54). Quite the opposite, both Na+-montmorillonite and citric acid in the whey protein matrix resulted in the development of films with less thermal stability, low tensile strength, and Young's modulus, while increment in elongation at break might be attributed to the plasticizing effect of citric acid (53). Oymaci and Altinkaya (55) added zein protein nanoparticles in the whey protein and sodium caseinate films. Inclusion of zein nanoparticles in the matrix of dairy protein and developed edible films noticeably enhanced moisture barrier and other physicomechanical properties. However, in the case of whey protein isolate-based films significant decrement was observed in the moisture barrier and fractional free volume. Qazanfarzadeh and Kadivar (56) also accentuated noteworthy improvement in the mechanical, moisture as well as gas barrier properties of milk protein based composite films encumbered with nanocelluloses. The developed edible films encumbered with nanocellulose at a 5% concentration level possessed high tensile strength, Young's modulus, water vapor permeability, solubility, and lower elongation at break. Additionally, no further improvement in water vapor permeability was observed with an increase in nanocellulose concentration but a decrement in tensile strength, Young's modulus, and solubility of the films was reported. Agudelo-Cuartas et al. (57) incorporated nanoemulsions of alpha-tocopherol and natamycin as bioactive ingredients in whey protein films. And it was found that developed films had shown noticeable increase in water vapor permeability, an excellent barrier to ultraviolet radiations as well as exhibited superlative antioxidant property as well as antimicrobial activity against *Candida albicans, Penicillium chrysogenus*, and *Saccharomyces cerevisiae*. Akin to this study Alizadeh-Sani et al. (58) employed rosemary essential oil and nanoparticles of zinc oxide in sodium caseinate-based films. Significant improvement was noticed in moisture and gas barrier properties of the developed films along with enhanced strength and flexibility. On the other hand, the moisture barrier properties of these active films decreased to a greater extent. An eco-friendly biodegradable film was prepared using calcium caseinate containing extract of *zingiber officinale* and Persian gum as active materials. These investigations suggested that incorporation of the active material as extract of z*ingiber officinale* significantly improved elongation at the break, tensile strength, and enhanced water solubility which further improved moisture barrier properties (59).

### Barrier properties

Excellent barrier properties of milk protein-based coatings/ films are attributed to the presence of polar amino acids in the chain of milk protein, which makes the films protective against non-polar molecules, especially oxygen, therefore, casein-based films serve as a boon for packaging the oxygen-sensitive food products (60). But the major limitations of these casein-based films are the intrinsic protein hydrophilicity due to which these films possess high sensitivity to moisture, which lowers the mechanical properties and barrier properties, which can otherwise be overcome by the incorporation of different types of active ingredients in these films (23, 61).

Whey protein films confine the rate of condensation of water vapors in packages containing cut and whole fruits and vegetables in a very efficient manner, therefore, inhibiting microbial spoilage. Hence, controlled conditions of relative humidity and the use of different plasticizer types significantly improve the moisture permeation properties of milk protein films (23).

Different research studies reveal that whey protein-sourced films/coatings have relatively reduced oxygen permeability which makes them suitable for coatings/film materials used for packing oxygen-sensitive commodities (62, 63). The moderately low oxygen permeability of whey protein-based films/coatings improves the quality of oxygen-sensitive foods by preventing oxidative damage to lipid components as well as reducing deterioration by aerobic microorganisms as evidenced in colored food products, confectionery items, fried foods, oilseeds, fruits and vegetables (23, 62, 64). Whey protein films/coatings appear to have a greater oxygen permeability in comparison to the films developed from animal and plant protein sources, such as collagen, gluten, zein, and genistein. These qualities of milk protein films give food a smooth, glossy surface, which gives protection against flavor and aroma losses as well as reduces moisture migration thereby preventing wilting (65).

### Antimicrobial properties

Whey protein isolate films are excellent carriers of various active ingredients viz. antimicrobials, and spices which further improve the permeation as well as other functional attributes of these films/coatings (66). Studies report that whey protein-sourced edible films are the superlative carriers of bioactive constituents viz. essential oils, bacteriocins (nisin and natamycin), plant extracts, probiotics, etc. (67, 68). The development of active packages containing natural antimicrobials is trending nowadays as per the increased demand of consumers for natural food ingredients.

Whey protein coatings/films show superb compatibility with different antimicrobial compounds such as sorbates, benzoates (p-aminobenzoic acid), lactates (sodium lactate), e-polylysine, enzymes (lysozyme and lactoperoxidase), essential oils, and bacteriocins both in terms of carrying as well as delivery/release of these antimicrobial compounds to improve the functional attributes of films (66). An active coating/film was developed using whey protein and propionic, benzoic, sorbic, lactic acid, lysozyme, and liquid smoke which showed preventive activity against the growth of different food spoilage bacteria, yeast, and mold in coated food materials. Whey protein-based active films incorporated with benzoic acid exhibited maximum inhibitory action against *Salmonella sp*. and *Escherichia coli* (64). Akin to the above study, the antimicrobial efficacy of whey protein-based films augmented with 1–2.5% essential oils of thyme cinnamon, and cumin on freshly sliced red meat was studied. It was revealed that the highest inhibition was noticed on surfaces of sliced meat in thyme essential oils incorporated with whey protein films. Furthermore, for the whey protein films containing significant doses of essential oils, there was a considerable drop in the total viable bacterial population during storage (69).

## Applications of milk protein based active edible packaging

Edible films/coatings are layers that protect food against adverse environmental conditions during storage and distribution and also act as barriers to mass and water transfer and so can be utilized to extend the shelf life of food products (70). Recently, the demand for edible, biodegradable, renewable, and non-toxic food packages is increasing among consumers (71). Film /coating preserves food properties including texture, color, nutrition, and microbiological. Proteins from different food sources are suitable materials for producing edible and biodegradable coating and films. Owing to their commendable functional properties, milk proteins not only work as a major ingredient in food but also can add interesting characteristics to edible films. Edible films manufactured from polymers are applied on fruits and vegetable surfaces (27). Films based on milk proteins (whey or casein) seem a very promising approach to improve and preserve the quality of food during storage as they offer a tremendous advantage over other conventional packaging systems. Along with being environmentally friendly, milk protein-based coating can provide additional benefits by incorporating active/ functional agents to them (2). Milk proteins have received a lot of attention in the past few years for delivering and protecting nutraceuticals including vitamins, essential oils, probiotics, antioxidants, bioactive peptides, and so on. Using milk proteins to encapsulate nutraceuticals aids food scientists in developing functional foods with beneficial health impacts. Milk proteins as nanocarriers have an indisputable significance in nanobiotechnology, where particles smaller than 1,000 nm are used. Milk proteins are studied because of their ability to entrap nutraceuticals and medications, protecting and transporting them till they reach their destination (72). Nanogel casein particles are prepared by using a crosslinking enzyme transglutaminase. These nanogels exhibit better stability against coagulation due to heat treatment. These nanogels could further be for encapsulation of various bioactive substances owing to their better sustainability in the acidic environment inside the stomach and also because of their sustained release at the target site (73). For instance, probiotics namely *Lactobacillus paracasei* ssp. *paracasei* F19 and *Bifidobacterium lactis* Bb12 were encapsulated in microcapsules obtained from gelled casein by transglutaminase enzyme. On further incubation of encapsulated and non-encapsulated probiotics in simulated gastric conditions (at pH 2.5 and 3.6), in the absence of pepsin, a shielding effect was revealed because of microencapsulation (74). Milk protein-based films are mechanically more adaptable and would better resist processing, storage, and end-use environment and provide a new alternative to commercial applications. Edible film/coating as a recent sustainable packing system offers significant potential to work as an effective barrier between the environment and food surface to ensure better safety and quality. Recently, whey protein (WP) has gained popularity among researchers as a most promising edible polymer in the food packing industry due to its tremendous functional properties, safety, and biodegradable nature. Whey protein is utilized in two forms as WP isolates and WP concentrate in the preparation on coating and films (23). Whey film is a dry polymer having a three-dimensional gel-like structure and is odorless, colorless, transparent, and flexible and possess outstanding barrier and mechanical properties as compared to other protein-based polymers and polysaccharides. WP-based coating is successfully reported as a vehicle of active agents like antioxidants, probiotics, antimicrobials, etc. without altering the native property of films and adding potential value to subsequent commercial applications (75). The latest food applications of milk protein-based film/ coating are presented in Table 2. Whey protein is an industrial by-product after paneer and cheese preparation and owing to its tremendous functional and beneficial properties, it has been successfully applied in food products as a base material for edible film/ coating. In the recent era, technological advancements in milk protein-based edible film processing, the addition of active material for improving functional characteristics have increased their potential application in the packaging of food materials (fruit, vegetables, meat products). As reported in Table 2, milk protein (whey or casein)-based coating/ films possessed significant antimicrobial properties against aerobic mesophilic bacteria, psychrotrophic bacteria, *Escherichia coli*, lactic acid bacteria, *Staphylococcus aureus, Bacillus cereus*, etc. along with better retention of physical parameters such as color, texture, organoleptic quality including increased shelf life. Another advantage of milk-based proteins over petroleum-based coating /films is their biodegradable and eco-friendly nature. So it can be utilized as a greater alternative to the already existing non-biodegradable materials extensively used in the food industry (109).

**Table 2:** Food applications of milk protein-based active edible packaging systems.

**Milk protein + active ingredient**	**Food products**	**Applications**	**References**
Whey protein (WP) as base material		
WP isolates + essential oil (Lemon or lemongrass)	Fresh cut pears	Better texture, firmness polyphenolic and flavonoids	(76)
WP concentrate + essential oil (rosemary)	Fresh spinach	Antimicrobial activity, better chlorophyll, and weight retention,	(77)
WPI + sodium montmorillonite nanoparticles + sodium metabisulfite	Eggs	Better retention of color and quality	(78)
WP concentrate + *Fucus vesiculosus* ethanolic extract	Poultry Meat	Antioxidant property, better physical properties	(79)
WP + pectin	Roasted peanuts	Antioxidant and antimicrobial properties, better water, and color retention	(80)
W P isolate/furcellaran (FUR) + *Borago officinalis* L.	Smoked pork ham	Darker in color, antioxidant and antimicrobial property	(81)
WP + cassava starch Whey protein + whey protein isolate + starch + RPE (rambutan peel extract) + cinnamon oil	Salami	Antibacterial activity, antimicrobial against *E. coli, S. aureus* and *B. cereus*	(82)
WP concentrate + Carboxy methyl cellulose + glycerol	Sunflower seed kernels	Antioxidant property and better retention of color	(83)
WP + chitosan + cranberry or quince juice	Fresh cut turkey pieces	Antimicrobial activity against *E. coli, S. typhimurium*, and *C. jejuni*	(84)
WP + immunoglobulin	Nisin	Preservation and better release of *Igs*	(41)
WP concentrate + Essential oil blend of *Cinnamomum cassia +Cinnamomum zeylanicum+ Rosmarinus officinalis* [1%, 2%, 2.7%, and 5% (w/w)]	Salami	Antioxidant property, hydrophobic	(45)
WP nanofibrils based edible coatings + glycerol (Gly) + trehalose (Tre)	Fruits and vegetables fresh cut apples	Hydrophobic and antioxidant activities, better texture, reduced degradation of total phenolic compounds, browning and product weight loss	(85)
WP + lactoperoxidase system (alginates)	Chicken thigh meat	Antimicrobial activity against *Pseudomonas aeruginosa, Enterobacteriaceae and* total aerobic mesophilic bacteria	(86)
WP concentrate + essential oil (*Origanum virens*)	Meat (Portuguese sausages)	Better color, antioxidant and antimicrobial property	(87)
WP isolate coating + Ginger + chamomile essential oils	Rainbow trout filets	Antimicrobial activity against *Pseudomonas*, lactic acid bacteria, psychrotrophic bacteria and total aerobic mesophilic bacteria	(88)
WP isolate + essential oil obtained from *Lavandula angustifolia* nanoparticles	–	Sustained release, better antimicrobial activity	(89)
WP isolate + MAP or vacuum package	Thawed bigeye Tuna chunks	Antibacterial activity, better biochemical and physical quality	(90)
WP nanofibrils based edible coating with titanium oxide nanotubes	Refrigerated meat	Resistance to oxidation, enhanced antimicrobial activity, enhancement in shelf life of meat held at lower temperature	(91)
WP isolate, extract of *Borago officinalis* + Furcellaran	Smoked and vacuum-packed Ham	Antioxidant potential improved dramatically, water activity lowered, storage life extended	(81)
WP isolate + glycerol coatings in conjugation with Poly(ε-caprolactone) (PCL) nanofibers and Nettle leaf extract	Fish filets	Decreased the population of spoilage bacteria, enhanced antioxidant potential, promotion of storage life	(92)
WP isolate + natural extract (*Laurus nobilis* L. or *Salvia officinalis*)	Cooked meatballs	Antioxidant property and color stability	(93)
**Casein as base material**			
Sodium caseinate + essential oil (Ginger)	Chicken breast filet	Significant antimicrobial property against total aerobic psychrophilic bacteria	(94)
Caseinate/zein + Curcumin.	Food	Antioxidant property and red yellow color	(95)
WP solutions or calcium caseinate + CMC (carboxymethyl cellulose)	Apple and potato slices	Reduced browning (enzymatic), better antioxidant, improved texture	(51)
Casein hydrolysate + WP isolate and; WPI + casein hydrolysate + oolong tea.	Beef steak and catfish filet	Antimicrobial property and a significant reduction in protein oxidation	(96)
Casein hydrolysate + WP isolate	Beef steak	Antioxidant property	(97)
Casein and WP concentrate.	Cheddar cheese	Better organoleptic quality and shelf life along with superior oxygen permeability	(42)
Casein hydrogel + Transglutaminase + Vitamin B12	–	Reduced the time required for gelation, higher degree of fractal pattern, sustained release of vit. B12	(98)
Casein-γ-polyglutamic acid hydrogels + transglutaminase + Vitamin B12 + Aspirin	–	Excellent drug delivery kinetics	(99)
Casein micelle and glyceraldehyde as crosslinker + Anticancer medicine		Superb compatibility, good encapsulation efficiency, very less seepage of drug, swift medicine dissolution in reaction to pH	(100)
Casen + calcium phosphate microspheres + Ibuprofen, vitamin B5 and docetaxel	–	Enhanced drug release characteristic with very less damage to the cells	(101)
Casein + Hyaluronic acid + Hydrophilic drugs	–	No *in vitro* cytotoxicity was observed, enhanced compatibility	(102)
Sodium caseinate + pectin hydrogels + Fish oil	–	Imparted resistance to oxidation, thereby imparting stability to fat rich meat products	(103)
Microspheres of sodium caseinate + low methoxy pectin hydrogel + Fish oil with ω – 3 fatty acid 312mg/g	–	Excellent defiance against oxidation, in addition to it, increased digestibility under simulated gut conditions in comparison to non-encapsulated particles	(104)
Sodium caseinate coating Gallic acid and rosemary oil	Fennel seeds	Escalated stability against oxidation with a decrease in water transferability	(105)
Sodium caseinate coatings incorporated with *Lactobacillus plantarum*	Grapes	Were helpful in maintain acidity and emerged as a better subsidiary method for bio preservation of grapes	(106)
Sodium caseinate + Arabic gum with lemon grass and cinnamon essential oil	Guava	A decree in polyphenol oxidase and peroxidase activity, higher antioxidant potential, superior holding ability of ascorbic acid, flavonoids and phenols	(107)
Calcium caseinate and/ or whey protein-based coating with carboxymethyl cellulose	Apples and potatoes	A boost in antioxidant ability, prevented browning	(108)

## Future prospects

The innovation in biodegradable package sources as a replacement for the plastic package is a real beginning in the food industry. Researchers and producers are focused on the production of coating material that is eco-friendly, with no waste, economical, easily available, and natural. Work is going on functionalizing these coating/ films and making them active/ intelligent packages. The same can be achieved by two pathways, either by the design of control release of bioactive material or package design with sensor alarms to alert the customer about changes in product properties. Whey protein-based film/ coating is a better alternative to existing conventional packaging material. Presently, despite being expensive, this is more valuable in terms of being eco-friendly and biodegradable in nature, suitable, vegetarian, and popular among customers (63).

The customer's demand for nutritive and healthy food from natural sources has created significance for the development of food having peculiar functional characteristics. Edible coating/films from natural ingredients can be an excellent way to elaborate the market of functional foods and can replace existing packaging from polymers in the food industry. Incorporation of milk-based protein, especially whey protein isolate/concentrate along with active materials into edible package film/coating, is a potential technique and can have application in numerous food products. Film/coating matrix, characteristics, and food preservation are majorly influenced by the type of ingredient material and formulation composition, and preparation technique. Further studies are required to evaluate the impact of whey protein-based coating on the shelf life and quality retainment of fresh coated/covered food products (27). Additionally, these coatings can be utilized as a vehicle of active material in functional food. Some of the studies have proved that whey protein coating application as a carrier for compounds like antimicrobial, antioxidants, various nutrients and still film properties (physical and mechanical) needs to be improved. Further techniques like blending, application of nanotechnology, and crosslinking of proteins by the enzymatic, physical, and chemical method can be helpful in the improvement of tensile strength, barrier property, and elongation at break characteristics of whey protein-based film/coating (110). A commercial potential advantage of WP coating as a carrier of bioactive compounds in fruits, vegetables, and cheese lies in their quality improvement and shelf-life extension. WP-based laminates (multi-layered) are also authentically approved for food storage. Thus, innovation in milk-based protein can be separated to make multi-layered films recyclable. It can be more helpful to maintain sustainability due to its recyclable nature than incineration as carried out in synthetic laminates as WP is a by-product of the cheese manufacturing food industry although, cost-effectiveness is the major driving force for WP processing against industry setbacks. Industrial applications of new innovative techniques are dependable on new scientific research aiming at the improvement in film-forming technology with better characteristics of film and product. Studies are also required to estimate the market value and long-term toxicity impact before implication in the market (23).

## Author contributions

All authors listed have made a substantial, direct, and intellectual contribution to the work and approved it for publication.

## Funding

Supported by a grant from the Romanian National Authority for Scientific Research and Innovation, CNCS—UEFISCDI, project number PN-III-P2-2.1-PED-2019-1723 and PFE 14, within PNCDI III.

## Conflict of interest

Author MT was employed by Food Research Department, Centre for Innovative Process Engineering (CENTIV) GmbH. The remaining authors declare that the research was conducted in the absence of any commercial or financial relationships that could be construed as a potential conflict of interest.

## Publisher's note

All claims expressed in this article are solely those of the authors and do not necessarily represent those of their affiliated organizations, or those of the publisher, the editors and the reviewers. Any product that may be evaluated in this article, or claim that may be made by its manufacturer, is not guaranteed or endorsed by the publisher.
